# Hybrid UNet transformer architecture for ischemic stoke segmentation with MRI and CT datasets

**DOI:** 10.3389/fnins.2023.1298514

**Published:** 2023-11-30

**Authors:** Wei Kwek Soh, Jagath C. Rajapakse

**Affiliations:** School of Computer Science and Engineering, Nanyang Technological University, Singapore, Singapore

**Keywords:** computed tomography perfusion imaging, ischemic strokes, lesion segmentation, magnetic resonance imaging, Vision Transformer

## Abstract

A hybrid UNet and Transformer (HUT) network is introduced to combine the merits of the UNet and Transformer architectures, improving brain lesion segmentation from MRI and CT scans. The HUT overcomes the limitations of conventional approaches by utilizing two parallel stages: one based on UNet and the other on Transformers. The Transformer-based stage captures global dependencies and long-range correlations. It uses intermediate feature vectors from the UNet decoder and improves segmentation accuracy by enhancing the attention and relationship modeling between voxel patches derived from the 3D brain volumes. In addition, HUT incorporates self-supervised learning on the transformer network. This allows the transformer network to learn by maintaining consistency between the classification layers of the different resolutions of patches and augmentations. There is an improvement in the rate of convergence of the training and the overall capability of segmentation. Experimental results on benchmark datasets, including ATLAS and ISLES2018, demonstrate HUT's advantage over the state-of-the-art methods. HUT achieves higher Dice scores and reduced Hausdorff Distance scores in single-modality and multi-modality lesion segmentation. HUT outperforms the state-the-art network SPiN in the single-modality MRI segmentation on Anatomical Tracings of lesion After Stroke (ATLAS) dataset by 4.84% of Dice score and a large margin of 40.7% in the Hausdorff Distance score. HUT also performed well on CT perfusion brain scans in the Ischemic Stroke Lesion Segmentation (ISLES2018) dataset and demonstrated an improvement over the recent state-of-the-art network USSLNet by 3.3% in the Dice score and 12.5% in the Hausdorff Distance score. With the analysis of both single and multi-modalities datasets (ATLASR12 and ISLES2018), we show that HUT can perform and generalize well on different datasets.

Code is available at: https://github.com/vicsohntu/HUT_CT.

## 1 Introduction

Restrictive blood flow can lead to ischemic stroke in the brain. Among all of the strokes, about 87% of them are ischemic strokes (Kuriakose and Xiao, 2020). It is often a result of an accumulation of thrombocytes along the path of the blood vessel, which prevents the mobility of the red blood cells. The hemoglobin's vital oxygen can no longer be supplied to the brain tissues. This leads to the death of the brain cells. Immediate identification and relevant treatments are required before it becomes irreversible. The main objective is to restore the blood flow to the affected region to prevent further damage to the brain tissues. According to Tsao et al. (2022), there is a global estimate of 3.48 million deaths due to ischemic stroke in 2020. It is, therefore, important to determine the region of the obstruction of the blood flow and accurately segment the outline of the ischemic stroke lesion.

Using non-contrast Computed Tomography (CT) imaging to evaluate ischemic stroke is fast and cost-effective. However, it is difficult to interpret the infarct core because of the subtle differences in texture and intensity. It is also difficult to interpret due to multiple artifacts, noise, and other tissue abnormalities. On the contrary, one can inject a contrast agent into the bloodstream to enhance CT imaging, also known as CT perfusion imaging, which highlights the blood perfusion in the brain.

Although non-contrast CT imaging can still provide important information about the lesion core, it does not offer more detailed information. CT perfusion imaging provides more distinct regions of ischemic stroke lesions, such as the infarct core and the penumbra, which is treatable and reversible. We can mitigate further damages by differentiating between the two regions. Non-contrast and contrast CT imaging analysis provides important information for further treatment.

In addition to CT imaging, magnetic resonance imaging (MRI) scans, such as T1-weighted (T1-w) images, are also commonly used to assess stroke lesions because they provide detailed anatomical information and better classify brain tissues. From the T1-w MRI scans, we can observe that the damaged tissue can appear as hypointense regions.

The imaging information obtained from CT imaging complements the information obtained from the MRI scans. The MRI technique is more sensitive to early infarction changes, so timely and accurate intervention can be provided. The downside of using the MRI is the availability of such service at the healthcare provider, while CT imaging is more widely accessible.

Segmenting CT perfusion lesions involves dividing the brain image obtained from CT perfusion scans into distinct regions, specifically focusing on identifying and outlining areas affected by an ischemic stroke. The first step is to identify and locate the affected area so that the clinicians can determine the traits of the lesion and provide the right treatment for the patient to slow down the damage. On the one hand, manually segmenting the lesions is frequently time-consuming. It requires expertise and sometimes produces inconsistent results. On the other hand, an automatic brain lesion segmentation method is more efficient in diagnosing and providing appropriate treatment for the condition of the brain.

The supervised deep learning methods have been improved over existing machine learning techniques in just a short period of time. One of the pioneering deep-learning methods, such as the UNet (Ronneberger et al., 2015), has been vastly popular in biomedical image segmentation due to its consistent and outstanding performance. It consists of a series of downsampling and upsampling convolutional layers, coupling with skip-connection between the layers to improve the learning stability.

Wong et al. (2022) proposed Subpixel Network (SPiN) that uses two networks to achieve state-of-the-art lesion segmentation on the ATLAS R1.2 dataset. The first network maps the input image to a high-dimensional embedding space at twice the input resolution. The second network then produces a confidence map using “subpixel” predictions. Four predictions from a 2 × 2 neighborhood represent each pixel in the output segmentation. The final output class for each pixel is obtained using a learnable downsampler to predict the contribution of each subpixel prediction in a local region corresponding to the pixel in the original resolution. This avoids using hand-crafted downsampling techniques such as bilinear or nearest neighbor interpolation. Prior works that addressed the challenges in ischemic stroke lesion segmentation include DUNet (Jin et al., 2019), CLCI-Net (Yang et al., 2019), and X-Net (Qi et al., 2019). DUNet extracts 2D and 3D features to improve the computation, while X-Net attempts to improve the long-range correlation of the regions by using a feature similarity module. On the contrary, CLCINet introduced another network to handle the segmentation of smaller parts of an organ. Recently, USSLNet improved the multi-scale convolution structure of Clerigues et al. (2019) and increased the receptive field to capture greater details.

While UNet has been highly successful in the field of biomedical segmentation, Transformer architecture has recently made its way to show good performance in both image classification and segmentation. The transformer architecture was originally introduced by Vaswani et al. (2017) as self-attention networks in the application of Natural Language Processing (NLP).

Apart from performing extremely well in NLP tasks when trained on a large corpus, the Transformer model has also performed well in computer vision. Although it works on sequences, we can apply a workaround by converting the images into patches represented in a sequence. The conversion is achieved by splitting the images into patches and mapping the patches through learnable network layers before providing them as input to the Transformer. Vision Transformer (ViT) has recently been used in medical imaging analysis, such as tumor-type classification of ultrasound images (Dosovitskiy et al., 2020). In brain tumor segmentation, there has been increasing research using ViT. However, convolutional neural network (CNN)-based U-Net remains a strong contender. ViT fits well for image classification tasks because it can learn long-range dependencies between pixels. However, they do not perform as well in segmentation tasks, which require the model to learn local and global information.

Hybrid architectures that combine ViT and U-Net have been proposed to address this. For example, the Swin-Unet architecture from Cao et al. (2021) uses a hierarchical structure to reduce the complexity of the ViT architecture and improve performance. However, this architecture is only designed for 2D scans because it is pre-trained on the ImageNet dataset. Tang et al. (2021) proposed a 3D Swin-Unet architecture with self-supervised learning to improve performance in brain tumor segmentation. There are other hybrid architectures such as the Mixed-Transformer UNet (MT-UNet; Wang H. et al., 2021), the Transformer Brain Tumor Segmentation (TransBTS; Wang et al., 2021a), and the UNet Transformer (Unetr; Hatamizadeh et al., 2022). These architectures use multiple transformers for the bottleneck to reduce the size of the ViT and the overall complexity. These hybrid architectures have shown promising results in brain tumor segmentation but there is still a lack of research on ischemic stroke segmentation.

Both MRI and CT perfusion scans are commonly used in brain lesion segmentation. In this work, we compare our proposed method HUT, with other state-of-the-art methods using MRI and CT perfusion datasets. We only utilize a single-modality T1-weighted dataset for the MRI scans, namely the Anatomical Tracings of Lesion After Stroke (ATLAS) R1.2 dataset. The brain tissue may appear darker for the damaged or dead brain tissue than the healthy brain tissue. This is due to a lower signal strength produced by inactive brain tissue. In contrast to MRI scans, we use multiple image modes in the CT perfusion dataset. Multi-modal images provide more diverse information on the brain tissue, which helps enhance analysis, diagnosis, and segmentation performances. The CT perfusion dataset we employ is the Ischemic Stroke Lesion Segmentation (ISLES) 2018 dataset. The dataset comprises images with three different parameters, namely the mean transit time (MTT), cerebral blood flow (CBF), and cerebral blood volume (CBV). An observation of a persistent MTT, decreased CBF and reduced CBV signify that it could be an infarct core. Observing persistent MTT, slightly reduced CBF, and near-mean CBV implies that it could be an ischemic penumbra that can still be treated. Although CT perfusion is a valuable tool for detecting and outlining acute ischemic stroke lesions, it does not have the same spatial resolution as MRI. Therefore, CT perfusion may not be as accurate as MRI in identifying the infarct core and penumbra.

Small lesions often occur in ischemic stroke, and segmenting them becomes a real challenge when using convolutional neural network (CNN) architectures. CNN models obtain global features through the aggregation operation of the convolution and pooling. A reduction of the spatial resolution can result in a loss of information about the smaller features of the images. This is why most CNN architectures may miss the detection and outlining of the smaller lesion, leading to misdiagnosis of the medical condition. On the contrary, the Vision Transformer (ViT) performs better than its CNN counterpart because it captures long-range and short-range correlations in sequence data using a self-attention mechanism. However, the ViT architecture requires a lot of data to train (Dosovitskiy et al., 2020). Similar to the application of NLP, the images are transformed into patches and arranged sequence data to be used for the model.

Our approach differs from existing hybrid systems such as UNETR, TransBTS (Wang et al., 2021a), TransUnet (Chen J. et al., 2021), and STHarDNet (Gu et al., 2022). TransBTS places the transformer at the bottleneck of the UNet architecture. Similarly, STHarDNet adds a Swin Transformer at the first skip connection of UNet and concatenates its output at the second layer before the final layer of the UNet decoder. TransUnet is similar to TransBTS but has an additional downsampling CNN layer at the transformer's output. UNETR utilizes CNN layers at the output of the skip connections and concatenates the output sequence representation from the upsampled CNN layers with a decoder similar to UNet. In contrast, our architecture takes two patches of different sizes at the input and multiplies the attention map from the output of the cross-transformer at the UNet decoder. Additionally, we utilize self-supervised training for the CLS token at the output of the cross-transformer to enhance performance.

A two-fold approach is established to exploit the inter-correlation between the modalities and the intra-correlation between the voxels. First, we introduce the ViT with convolution layers to address lesion anomalies. Second, we present a self-supervised methodology to improve the convergence rate and the learning of the latent features.

In summary, we have made the following novel contributions to this work:

1. Introduce a Hybrid U-Net Transformer Segmentation system that performs state-of-the-art ischemic stroke segmentation on ATLASR12 and ISLES 2018 datasets. ATLASR12 contains one modality MRI (T1w), while the ISLES contain four CT perfusion images (CBF, CBV, MTT, and Tmax).
2. Our framework allows simultaneous self-supervised and supervised training on the UNet and Transformer networks.

## 2 Methods

### 2.1 Hybrid UNet transformer (HUT) architecture

The U-Net architecture uses convolution layers that provide an inductive bias to a system and increase the convergence rate, exploiting the local correlation between pixels via the kernels. On the other hand, the Transformer offers a long-range relationship between the tokens, represented mainly by the image patches. However, transformers are not data-efficient and require large datasets for the training to converge effectively. We also know that annotated data is costly and scarce in medical imaging. In light of the limitation, we introduce a hybrid network incorporating the merits of convolution layers and Transformers (Wang et al., 2021a; Wang H. et al., 2021). Moreover, we extract the information from the lower layers of the decoder of the UNet for the self-supervision learning of the CLS tokens.

**Figure 1:**
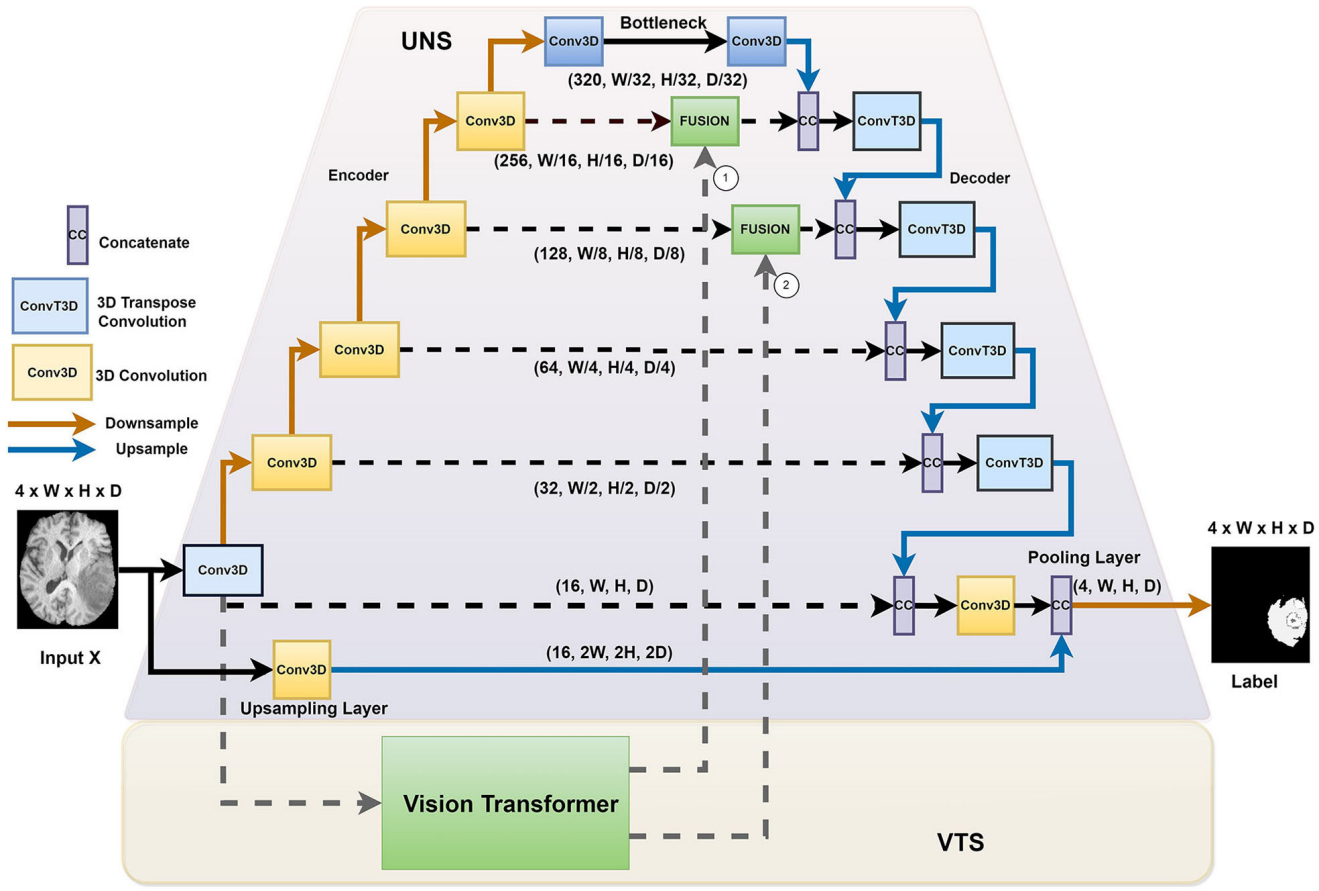
The HUT architecture consists of two separate stages: the UNet stage (UNS) and the Vision Transform stage (VTS).

Figure 1 illustrates the overall architecture of the network for ischemic stroke segmentation, which consists of two stages, namely the UNet stage (UNS) and the Vision Transformer stage (VTS). First, we incorporate the transformer blocks parallel to the U-Net structure. We instantiate a voxel embedding, a local Transformer, a position embedding, and a global transformer within the transformers module. The small patch transformer acts on the smaller voxel patches, whereas the large patch transformer acts on the larger voxel patches. The small patch transformer gathers information about local features, whereas the large patch transformer gathers information about the overall image. The traditional U-Net has better convergence, mainly due to inductive bias from the CNN architecture. We adopt a hybrid architecture to improve the voxels' local and global correlation and achieve faster convergence than training a pure transformer-based U-Net. In our proposed architecture, the transformers operate on each skip connection. By providing essential attention to important regions, we observed that the system performs better when incorporating transformers at the upper layers of the UNet.

We introduced the HUT architecture to address the shortcomings of the convolutional networks and transformer networks. Due to the inductive bias of the convolutional network, the UNet model is very data-efficient as it generalizes image features well. The transformer network uses self-attention to correlate long-range dependencies between image tokens. However, the transformer architecture requires large amounts of data to train to generalize well. The HUT architecture incorporates the VTS parallel to the UNS to overcome these limitations. Through this combination, we observe that UTP can capture long-range relationships between different patches, while VTS is trained more efficiently with the aid of the UNet.

### 2.2 The UNet stage (UNS)

We adopt the conventional UNet structure. As illustrated in Figure 1, we proposed introducing an extra upsampling layer to the first skip connection at the UNet stage via a conventional transpose layer with a kernel of 5 and stride of 2, followed by a pooling. It allows the model to capture smaller details while increasing the receptive field compared to the implementation of the first passthrough skip connection. By combining the UNS with VTS, the transformer can effectively leverage the advantage of an inductive bias of UNS and allow the learning to converge faster. In other words, compared to the traditional training of the transformer that requires a large amount of data, the training of the VTS is now more data-efficient with the help of the UNS. It is also important to note that the fusing of the transformer's output occurs at the lower layers of the decoder of the UNet. Fusing at higher layers incurs higher computation complexity and, naturally, does not help in the training efficiency of the transformer.

### 2.3 The Vision Transformer stage (VTS)

The original transformer architecture was introduced to address the long-term forgetfulness of the LSTM. It has been very effective on many natural language processing tasks. In contrast, a Vision Transformer (ViT) is an alternative form of the transformer that is developed especially for classifying and outlining objects in the images. Similar to the tokens in the NLP, we divide the images into smaller patches and map them into a sequence of patch tokens.

In most NLP applications, an extra token called the CLS (Classification token) is introduced to the input sequence to aggregate the relationship between the tokens. Training it with a label allows the Transformer to perform classification tasks such as sentiment analysis or text classification (Devlin et al., 2018). The transformer calculates the CLS token's representation by transversing every token's hidden states in the input sequence. As the tokens pass through the transformer's encoder layers, information is aggregated and represented within the CLS token. This aggregation and compressed representation of data is important to classification. Therefore, training the CLS token against the label can guide the model to learn a particular classification task. A couple of ViT variants use the CLS token for the task identification objects. A system that learns to identify objects performs well in the segmentation task.

The VTS comprises two layers: a self-attention layer and a feedforward layer. The self-attention layer allows the model to learn the importance of various tokens and build this part of the information into the representation. A position embedding is included within each token to avoid excluding important information about the relative position between the image patches. Most of the information will be combined into a representation in the CLS token and then used to train against the ground truth label. The CLS token will be used for prediction during the inference of a classification task.

**Figure 2:**
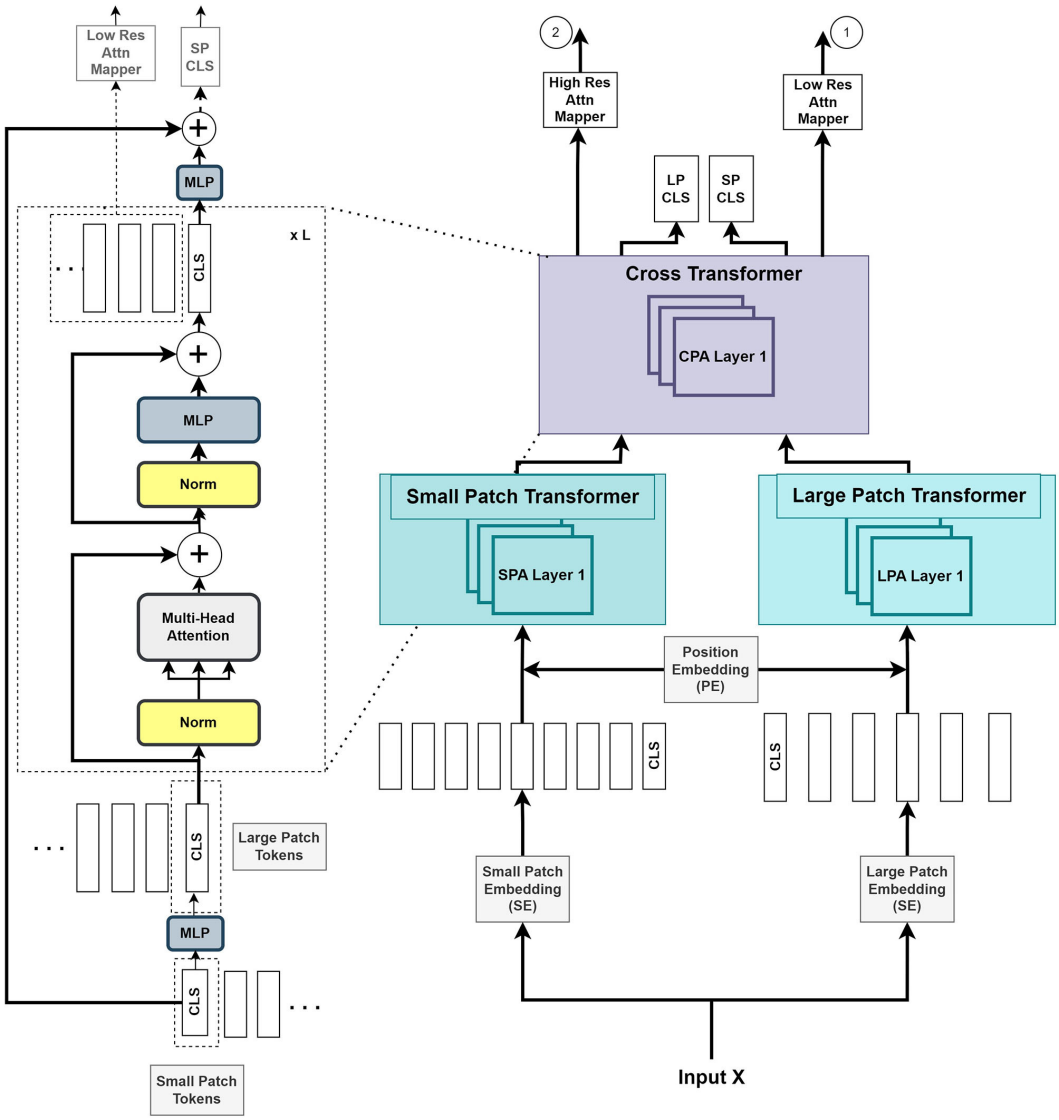
The Vision Transformer stage (VTS) comprises two branches of transformers for the small patches and large patches of the image. The last stage of cross-transformer is to relate the tokens from the output of the earlier branches.

As illustrated in Figure 2, we introduce two parallel transformer networks to process small and large image patches of two different resolutions (Chen C.-F. R. et al., 2021). Each voxel in the images is mapped to a vector embedding. The small patch attention (SPA) operates on tokens of voxel embedding to compute the self-attention correlation. The large patch attention (LPA) works on the coarser resolution. We denote *X* as the input voxel data such that *X*∈ℝ^*W*×*H*×*D*×*C*^, where *W, H*, and *D* are the dimensions of input, and *C* is the channel length. *f*_*s*_ and *f*_*l*_ are the linear network mapper functions that transform the input to the same dimension and *R*_*s*_ is the residual operation such that *R*_*s*_(*f*(*X*)) = *f*(*X*)+*X*.

The large patch embedding output $Y^{LA}\in {\mathbb{R}}^{\left(W/k\right)\times \left(H/k\right)\times \left(D/k\right)\times C_{L}}$, with dimensions *W*/*k, H*/*k, D*/*k*, are the voxel patches from embedding function with patch size *k* with *k*>*p*, and *C*_*L*_ is the new embedding channel length. Similarly, the small patch embedding output $Y^{SA}\in {\mathbb{R}}^{\left(W/p\right)\times \left(H/p\right)\times \left(D/p\right)\times C_{S}}$, with dimensions *W*/*p, H*/*p, D*/*p*, are the voxel patches from embedding function with patch size *p* and *C*_*S*_ is the new embedding channel length.

The following is a description of the operation of the HUT architecture:


(1) $$\begin{array}{l} \begin{array}{cc} S & =\text{SE}\left(X\right)+\text{PE}\\ Y^{SA} & =f_{s}\left(R_{s}\left(\text{LN}\left(\text{SPA}\left(R_{s}\left(\text{LN}\left(S\right)\right)\right)\right)\right)\right) \end{array} \end{array}$$

(2) $$\begin{array}{l} \begin{array}{cc} L & =\text{LE}\left(X\right)+\text{PE}\\ Y^{LA} & =f_{l}\left(R_{s}\left(\text{LN}\left(\text{LPA}\left(R_{s}\left(\text{LN}\left(L\right)\right)\right)\right)\right)\right) \end{array} \end{array}$$

where *S* and *L* are the outputs of voxel patch embedding of small and large patches, respectively. *Y*^*SA*^ and *Y*^*LA*^ denote the outputs from SPA and LPA blocks, respectively. The learnable small and large voxel patch embedding and position encoding function is denoted as SE, LE, and PE, respectively, and LN as layer normalization operation. The combined output *Y* of the two transformers is given by


(3) $$\begin{array}{l} \begin{array}{cc} Y & =\text{Concatenate}\left(Y^{SA},Y^{LA}\right)\\ Z & =R_{s}\left(f\left(\text{LN}\left(\text{CPA}\left(R_{s}\left(\text{LN}\left(Y\right)\right)\right)\right)\right)\right) \end{array} \end{array}$$

where *Z* denotes the output of the cross-transformer module, which consists of the cross-patches attention (CPA) function.

Figure 2 illustrates that the VTS produces two attention maps that will be fused with UNS. Furthermore, the CLS tokens will be used for self-supervised training with neither additional annotated nor extra unannotated data. We attempted a few options for the fusing mechanism between the UNS and VTS and found that the multiplication operation produces the best outcome. We merge the CLS tokens from the large and small patch transformers by mapping them to appropriate dimensions and exchanging them with other tokens. This is also known as the cross-attention of tokens (Wang et al., 2021b). We utilize the CLS token at each stage to exchange data between the tokens of the other branch. The CLS tokens can extract abstract details across the large and small patch tokens by relating the information between the patch tokens in another branch. Combining this additional information provides a better representation of the encoder output. We use the softmax function to transform the two final CLS tokens with linear layers to the output. A self-supervised training is introduced by matching the two probability distributions of the two CLS tokens. This method further improves the performance of the system.

### 2.4 Training the model

During the learning of the model, both the self-supervised training of the output of the ViT and the supervised training of the encoder-decoder structure of the UNet are trained concurrently with the training dataset. To ensure a low KL divergence between the probabilities of the outputs of the cross-transformer, we seek to minimize the cross-entropy loss between the CLS tokens. The self-supervised training at the CLS tokens ensures consistency between the probability distributions of the CLS tokens from the large and small patch transformers.

#### 2.4.1 Self-supervised loss function

Since the output of the classification layer from the large and small patch transformers should be similar in principle, we match the two output probability distributions from the CLS tokens through KL divergence. This is to ensure the consistency of the output.

The similarity between the probability distribution *p*_*CLS*_ of the small patch CLS token and the probability of the large patch CLS token, *q*_*CLS*_, is ensured through the cross-entropy loss function. We express the cross-entropy loss between the large patch CLS token probability output, *q*_*CLS*_, and the small patch CLS token probability output, *p*_*CLS*_ as:

(4) $$\begin{array}{l} L_{SS}=-p_{CLS}log\left(q_{CLS}\right) \end{array}$$

#### 2.4.2 Supervised loss functions

The motivation for a mixed segmentation loss of dice loss and cross-entropy is that dice loss handles class imbalance while cross-entropy loss allows a faster convergence in training. A weighted cross-entropy focusing on the minority class is well-suited for class imbalance datasets. Under supervised learning, combining a weighted sum of soft dice loss and cross-entropy loss forms the segmentation loss.

The cross-entropy loss measures the difference between the probability of predicted output $q_{c}^{i}$ and the ground truth of pixel *i* and the probability distribution $p_{c}^{i}$ of the class label *c*. We write the loss function as:


(5) $$\begin{array}{l} L_{CE}=-\frac{1}{N}\underset{c}{\sum }\underset{i}{\sum }p_{c}^{i}log\left(q_{c}^{i}\right) \end{array}$$

The soft dice loss at the output of the softmax function of the network is represented as follows:


(6) $$\begin{array}{l} L_{Dice}=\frac{1}{N}\underset{c}{\sum }1-\frac{2\sum_{i}q_{c}^{i}p_{c}^{i}}{\sum_{i}\left(q_{c}^{i}+p_{c}^{i}\right)} \end{array}$$

where *N* denotes the number of batches. With λ_*Dice*_ chosen empirically for the ATLAS lesion segmentation task and $q_{c}^{i}$ is the probability of a predicted class, $p_{c}^{i}$ is the probability of actual class at pixel *i*, we represent the total loss for the segmentation network as:


(7) $$\begin{array}{l} L=\lambda_{CE}L_{CE}+\lambda_{Dice}L_{Dice}+\lambda_{SS}L_{SS} \end{array}$$

where λ_*Dice*_, λ_*CE*_, and λ_*SS*_ are the weighting factors for the dice and self-supervised losses.

The λ_*Dice*_ and λ_*CE*_ are set as 0.5 and 0.5 for the lesion segmentation task in all the experiments. For λ_*SS*_, it works empirically better with the value of 1*e* − 4 for the ATLAS dataset. For the lesion segmentation task on the ISLES dataset, an empirical value of 1*e* − 5 is chosen for the λ_*SS*_ factor.

## 3 Experiments and results

In this section, we perform experiments with a single modality for ischemic stroke lesion segmentation with the ATLASR12 dataset and four modalities of CT perfusion scans with the ISLES2018 dataset.

We use the Dice score, HD95 score, IOU, Precision, and Recall as the evaluation metrics to evaluate the testing set. The Dice and HD95 scores are the more important metrics for lesion segmentation. We employ an equal weighting of soft dice and cross-entropy loss to train all the segmentation networks in these experiments.

### 3.1 Ischemic stroke lesion segmentation from T1-weighted MRI scans

In this section, we demonstrate experiments with the ATLAS R1.2 dataset.

The ATLAS dataset (Liew, 2017; Liew et al., 2018) comprises 304 T1-weighted MRI scans of stroke patients with corresponding lesion annotations. The data were manually annotated to identify the stroke lesions. It was collected from 11 research locations worldwide. The scans were then processed for privacy by smoothing and defacing. The remaining data contains 239 patient scans. To reduce the requirement of GPU memory, we cropped each 3D scan to a resolution of 160 × 160 × 192 and focused on relevant regions of the image. The ischemic stroke dataset contains very small lesions, which can make segmentation tasks difficult. To compare with the results in Wong et al. (2022), we used the same random data split of the ATLAS dataset that the authors had evaluated, with 212 train and 27 test subjects, with about 89% training subjects and 11% testing subjects.

In the paper of Wong et al. (2022), the authors qualify anomalies < 100 pixels as small lesions. We evaluated the performance of segmenting small lesions in a similar fashion. In the experiment, the same criteria for the evaluation of the task of small lesion segmentation were used.

All the segmentation networks in the experiments used equal weighting of soft dice loss and cross-entropy loss for the training.

The metrics used to evaluate the ischemic stroke lesion segmentation are Dice (Zou et al., 2004), HD95 (Cárdenes et al., 2009), IOU (Cárdenes et al., 2009), Precision (Udupa et al., 2006), and Recall (Udupa et al., 2006).

As illustrated in Table 1, our HUT method improves the mean Dice score (DSC) performance over the state-of-the-art SPiN (Wong et al., 2022) architecture by 4.84%. HUT gains the mean of 95th percentile Hausdorff Distance score (HD95) over SPiN by 40.7%.

**Table 1:** Comparison between mean and standard deviation (in parentheses) of dice score, HD95 score, IoU, precision, and recall of the ischemic stroke lesion segmentation by HUT against state-of-the-art methods with ATLASR12 dataset.

**Methods**	**Dice**	**HD95 (mm)**	**IOU**	**Precision**	**Recall**
UNet3D	0.665	13.947	0.523	0.765	0.614
(Ronneberger et al., 2015)	(0.186)	(15.756)	(0.175)	(0.240)	(0.192)
DUNet	0.548	22.809	0.404	0.652	0.521
(Jin et al., 2019)	(0.216)	(24.393)	(0.187)	(0.258)	(0.241)
ERFNet	0.670	13.262	0.522	0.818	0.609
(Romera et al., 2017)	(0.150)	(13.957)	(0.156)	(0.202)	(0.165)
UShape	0.673	13.714	0.530	0.777	0.628
(Clerigues et al., 2019)	(0.182)	(16.168)	(0.170)	(0.214)	(0.214)
USSLNet	0.694	12.563	0.545	0.763	0.682
(Jiang and Chang, 2022)	(0.130)	(13.113)	(0.142)	(0.195)	(0.151)
TransBTS	0.661	19.782	0.517	0.752	0.662
(Wang et al., 2021a)	(0.173)	(25.254)	(0.175)	(0.254)	(0.150)
UNETR	0.630	23.083	0.476	0.725	0.608
(Hatamizadeh et al., 2022)	(0.148)	(22.046)	(0.152)	(0.767)	(0.176)
CLCI-Net	0.599	20.802	0.469	0.741	0.536
(Yang et al., 2019)	(0.257)	(22.644)	(0.232)	(0.258)	(0.276)
X-Net	0.627	17.143	0.489	0.722	0.598
(Qi et al., 2019)	(0.216)	(15.897)	(0.204)	(0.208)	(0.264)
KiUnet	0.524	19.255	0.387	0.703	0.459
(Valanarasu et al., 2020)	(0.226)	(16.290)	(0.206)	(0.237)	(0.241)
SPiN	0.703	17.427	0.556	0.806	0.654
(Wong et al., 2022)	(0.129)	(19.469)	(0.142)	(0.123)	(0.182)
nnUnet	0.713	14.294	0.568	0.767	**0.707**
(Isensee et al., 2021)	(0.145)	(16.133)	(0.156)	(0.218)	(0.134)
HUT (ours)	**0.737**	**10.335**	**0.598**	**0.825**	0.706
	(0.127)	(10.074)	(0.144)	(0.172)	(0.153)

Bold values highlight the best performing values.

USSLNet performs close to SPiN for the dice score and outperforms SPiN for HD95. Furthermore, USSLNet is currently the state-of-the-art method on CT Perfusion dataset such as the ISLES2018 dataset. nnUNet outperforms both SPiN and USSLNet. It is currently the state-of-the-art method for Brain Tumor Segmentation (BraTS) dataset. However, HUT still has the performance advantage over nnUnet on the ATLASR12 and ISLES2018 datasets.

HUT performs much better than UNETR on the ATLASR12 dataset, with a 16.9% improvement in the dice score and a 24.5% improvement in HD95. It is 22.6% better than UNETR in dice score for small lesion segmentation. We compare the performance of another hybrid transformer Unet-based implementation and observe that HUT gains 11.5% in dice score over TransBTS. All other methods described in Wong et al. (2022) performed worse than HUT and are shown in Table 1. In terms of dice and HD95 scores for the task of lesion segmentation, HUT surpasses the performance of all other methods by a noticeable margin.

Table 2 shows HUT gains the dice score of small lesion segmentation over state-of-the-art SPiN by 18.6%. It improves the HD95 score of small lesion segmentation over SPiN by 42.6%. As for small lesion segmentation, our method indeed outperforms all other methods in Wong et al. (2022) by a larger margin.

**Table 2:** Comparison between performance metrics of the ischemic stroke small lesion segmentation by HUT against state-of-the-art methods with ATLASR12 dataset.

**Methods**	**Dice**	**HD95 (mm)**	**IOU**	**Precision**	**Recall**
**UNet3D**	**0.144**	**39.045**	**0.083**	**0.606**	**0.091**
**(Ronneberger et al., 2015)**	**(0.122)**	**(22.287)**	**(0.082)**	**(0.315)**	**(0.085)**
DUNet	0.265	26.730	0.180	0.377	0.264
(Jin et al., 2019)	(0.250)	(23.336)	(0.188)	(0.332)	(0.269)
ERFNet	0.401	17.268	0.262	0.574	0.406
(Romera et al., 2017)	(0.152)	(16.006)	(0.125)	(0.306)	(0.179)
UShape	0.321	15.472	0.205	0.545	0.270
(Clerigues et al., 2019)	(0.174)	(13.473)	(0.127)	(0.319)	(0.187)
USSLNet	0.408	15.496	0.274	0.553	0.460
(Jiang and Chang, 2022)	(0.185)	(13.379)	(0.155)	(0.306)	(0.225)
TransBTS	0.147	46.694	0.095	0.264	0.159
(Wang et al., 2021a)	(0.206)	(34.048)	(0.147)	(0.318)	(0.231)
UNETR	0.385	19.710	0.258	0.580	0.375
(Hatamizadeh et al., 2022)	(0.196)	(20.774)	(0.159)	(0.327)	(0.209)
CLCI-Net	0.246	22.884	0.178	0.417	0.215
(Yang et al., 2019)	(0.290)	(25.531)	(0.232)	(0.384)	(0.279)
X-Net	0.335	22.885	0.237	0.491	0.309
(Qi et al., 2019)	(0.274)	(22.294)	(0.221)	(0.340)	(0.292)
KiUnet	0.246	15.979	0.173	0.466	0.206
(Valanarasu et al., 2020)	(0.270)	(16.255)	(0.211)	(0.402)	(0.253)
SPiN	0.398	23.063	0.287	0.575	0.350
(Wong et al., 2022)	(0.274)	(20.764)	(0.229)	(0.332)	(0.272)
nnUnet	0.465	16.054	0.322	0.579	**0.515**
(Isensee et al., 2021)	(0.190)	(12.081)	(0.168)	(0.291)	(0.219)
HUTn (ours)	**0.472**	**12.630**	**0.327**	**0.634**	0.487
	(0.178)	(11.658)	(0.159)	(0.290)	(0.208)

Bold values highlight the best performing values.

**Figure 3:**
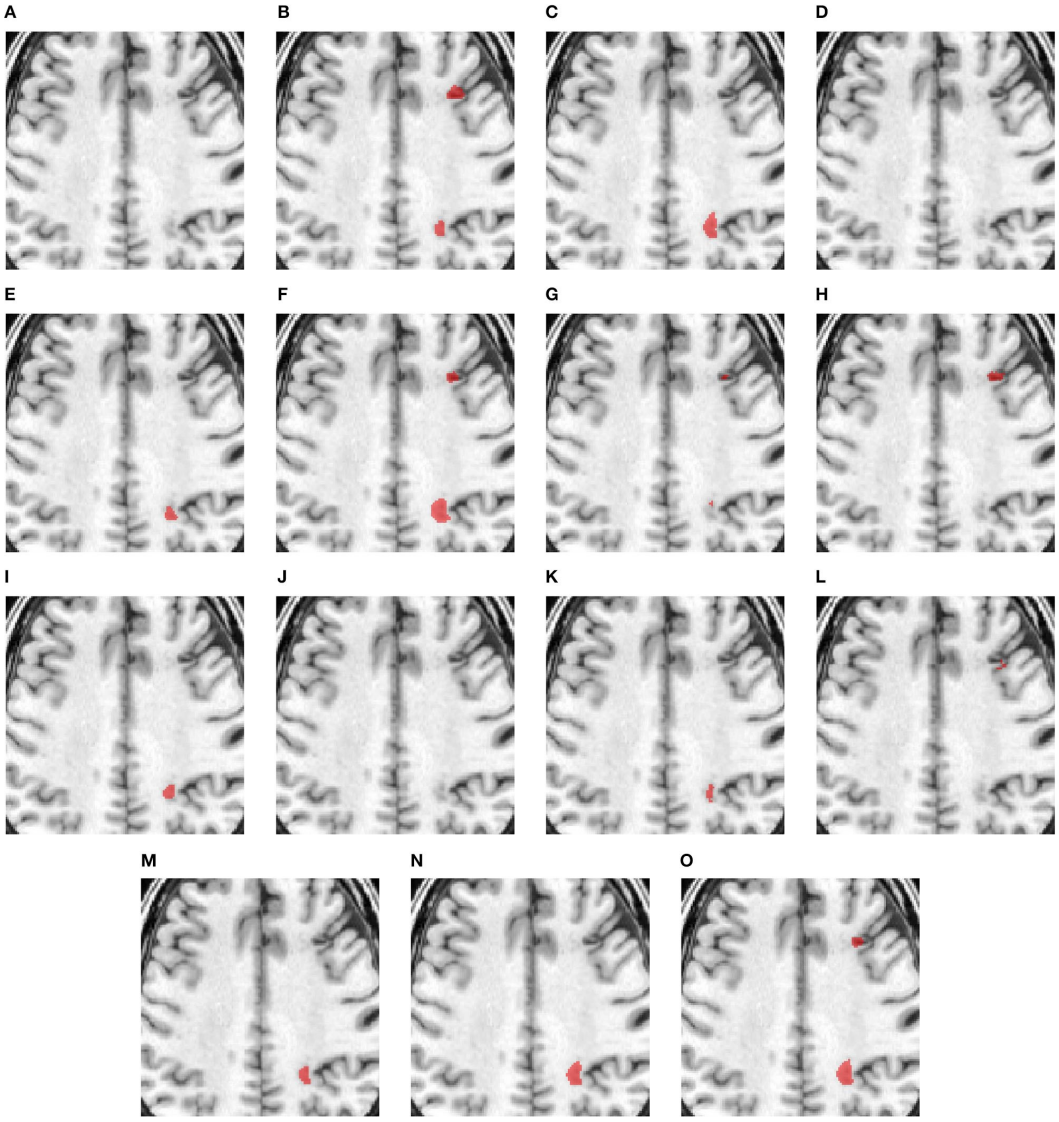
Illustration of small lesion segmentation of a representative subject 02 based on various methods. **(A)** T1w MRI scan, **(B)** ground truth, **(C)** UNet3D, **(D)** DUnet, **(E)** ERFNet, **(F)** UShape, **(G)** USSLNet, **(H)** TransBTS, **(I)** UNETR, **(J)** CLCI-Net, **(K)** X-Net, **(L)** KiU-Net, **(M)** SPiN, **(N)** nnUnet, **(O)** HUT.

Figure 3 compares the performance of various methods for predicting segmentation on a representative scan, including TRANSBTS, UNETR, SPiN, KiUnet, CLCInet, X-Net, and HUT. We also include methods like USSLNet, UShape, and ERFNet used in CT perfusion datasets. Most methods cannot accurately predict the two locations of small lesions, except for UShape, and HUT. Methods like SPiN, nnUNet, UNet3D, X-Net, USSLNet and ERFNet cannot detect the lesion at the upper region. Meanwhile, methods like DUNet, KiUNet, and CLCI-Net cannot detect any lesion.

**Figure 4:**
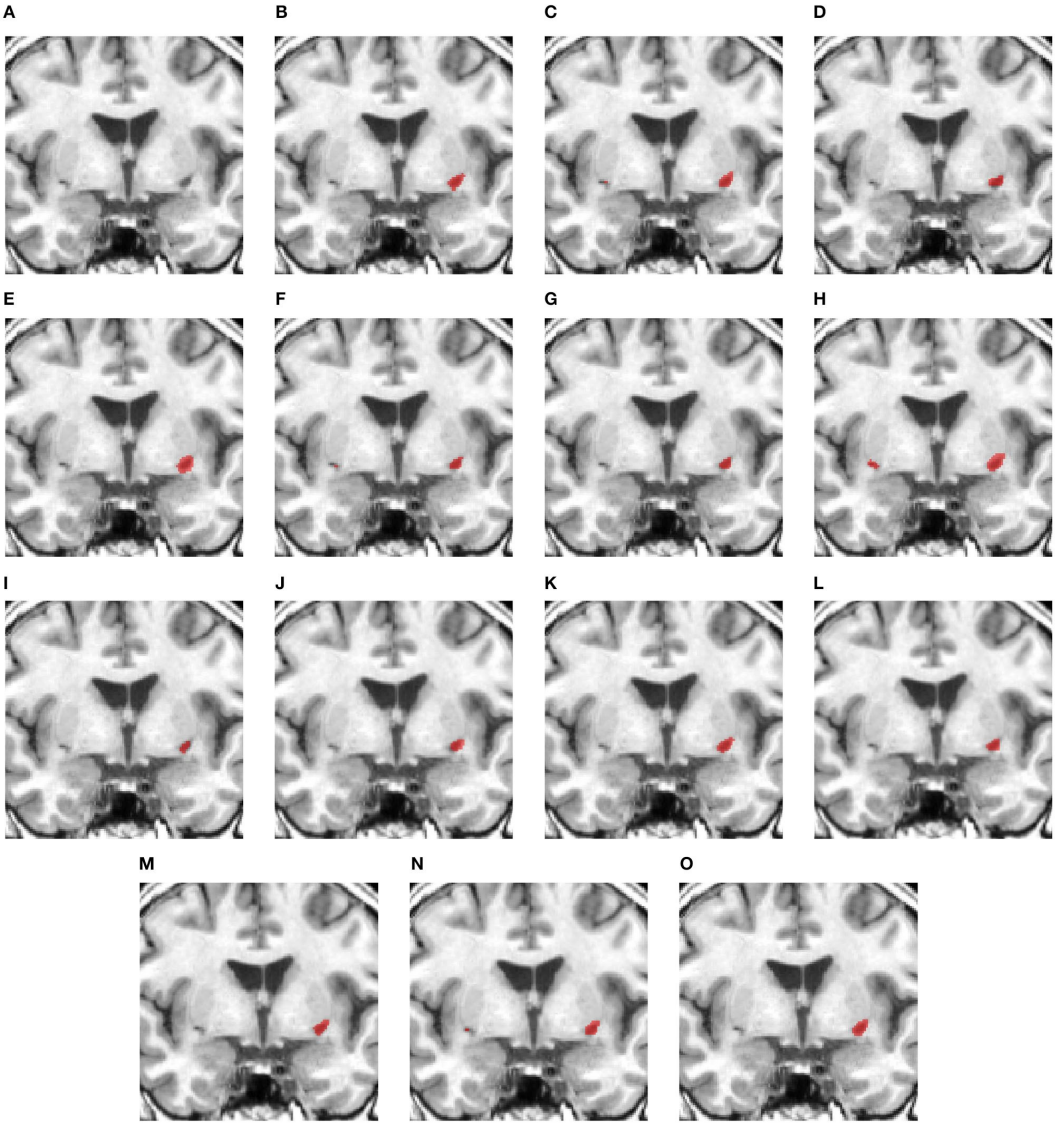
Illustration of small lesion segmentation of a representative subject 24 based on various methods. **(A)** T1w MRI scan, **(B)** ground truth, **(C)** UNet3D, **(D)** DUnet, **(E)** ERFNet, **(F)** UShape, **(G)** USSLNet, **(H)** TransBTS, **(I)** UNETR, **(J)** CLCI-Net, **(K)** X-Net, **(L)** KiU-Net, **(M)** SPiN, **(N)** nnUnet, **(O)** HUT.

Figure 4 shows a case with only a small lesion at this brain location. All methods can locate the right lesions, although nnUNet, UNet3D, UShape, and TransBTS incorrectly detect a lesion on the left side of the brain, yielding a false positive of a lesion.

**Figure 5:**
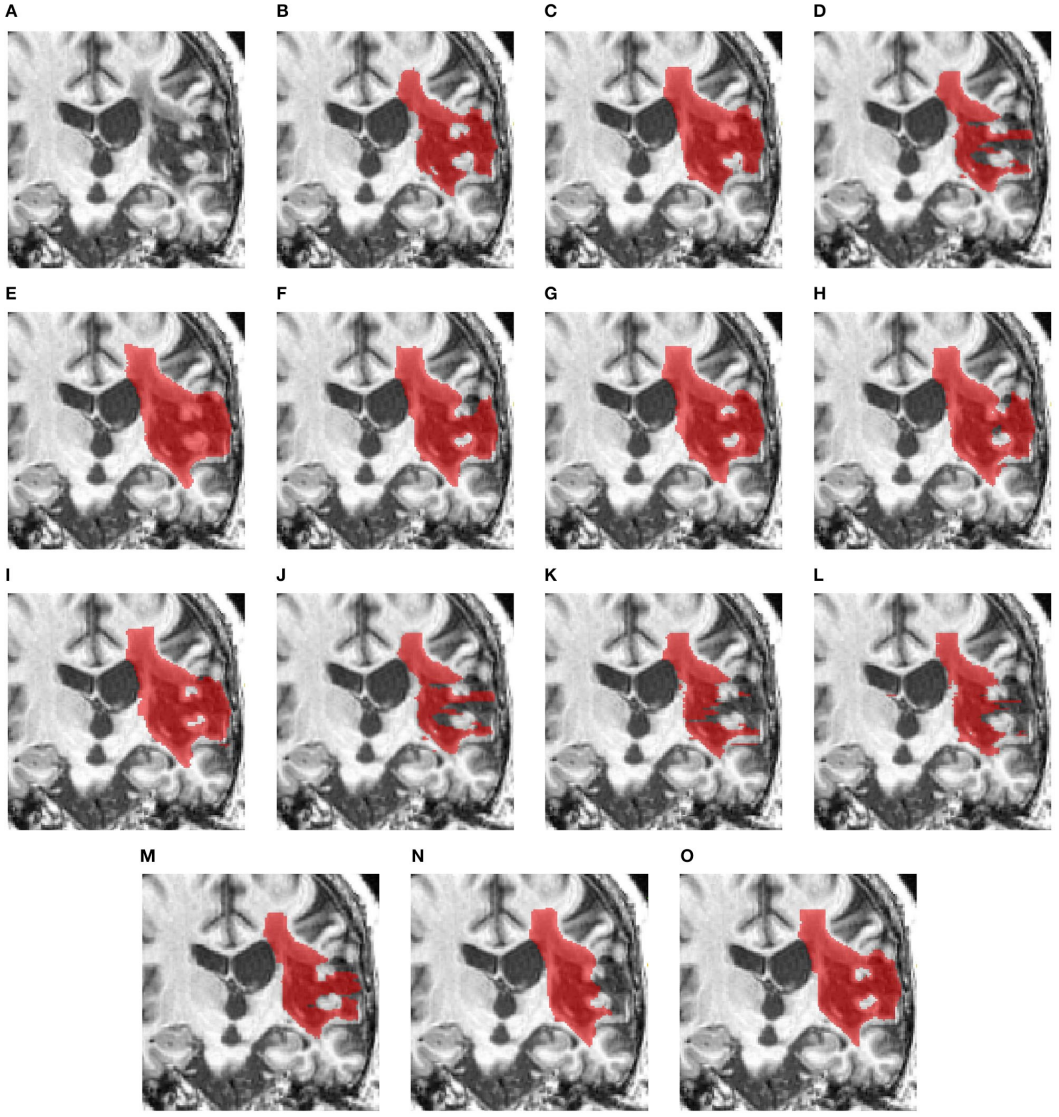
Illustration of large lesion segmentation of a representative subject 11 based on various methods. **(A)** T1w MRI scan, **(B)** ground truth, **(C)** UNet3D, **(D)** DUnet, **(E)** ERFNet, **(F)** UShape, **(G)** USSLNet, **(H)** TransBTS, **(I)** UNETR, **(J)** CLCI-Net, **(K)** X-Net, **(L)** KiU-Net, **(M)** SPiN, **(N)** nnUnet, **(O)** HUT.

Figure 5 examines a case where there is a large lesion. In this case, all methods can detect the region of the lesion. The difference between the segmentations is the shape of the lesion. USSLNet, UNETR, and HUT have the closest shapes, similar to the ground truth. CLI-Net, KiU-Net, X-Net, and nnUNet are more conservative in detecting the lesion on the right side nearer to the skull.

### 3.2 Ischemic stroke lesion segmentation using CT perfusion scans

For the second ischemic stroke lesion segmentation, we used the ISLES 2018 dataset (Cereda et al., 2016; Hakim et al., 2021), which consists of 94 CT perfusion scans. Each volume's width, height, and depth are 240 pixels, 240 pixels, and 2, 4, or 8 layers, respectively. The dataset has four CT perfusion modalities: CBF, CBV, MTT, and Tmax. A segmentation map of each volume consisting of two classes, namely, background and lesion, was manually annotated and curated by expert radiologists.

Data augmentation routines such as random affine of (0.75, 1.25) and rotation of 15° with a probability of 30% were conducted during training experiments. We ensured the same testing data were used consistently for all the experiments compared to existing techniques. The training and testing sets were randomly sampled about 90% of the total 94 subjects and 10% for testing. Furthermore, we ran and averaged five different runs for each method since the selected number of testing samples was small compared to the ATLAS dataset. Nevertheless, the variability of the dataset is large, and overfitting could be an issue in the experiments. We implemented a dropout of 25% and data augmentation to prevent overfitting. We used a learning rate of 3e-4 and a decay rate of 1e-7 on an Adam optimizer for all the experiments. The number of epochs for all the experiments was 1000.

Since the CT perfusion dataset has a limited amount of slices, i.e., 2, 4, or 8 for each subject, we attempt to use a chunk of slices to predict one slice of the segmentation map or alternatively use one slice of input to predict the output. There are four modality images for each slice. In the later section of the ablation study, we show the difference in performance for both approaches. Therefore, we modify the 3D model of HUT to adapt to the application in CT perfusion. We adopt the slice-by-slice 2D approach for the experiment, which yields a better performance for this dataset.

We compared with various methods like CNN-based UNet3D (Ronneberger et al., 2015), ERFNet (Romera et al., 2017), UShape (Clerigues et al., 2019), and USSLNet (Jiang and Chang, 2022), hybrid Transformer-based like TransBTS (Wang et al., 2021a), and UNETR (Hatamizadeh et al., 2022) networks. We also did an additional comparison with methods used on MRI datasets such as the ATLAR12 dataset. These methods' input channels are modified to take in the 4 modalities images.

Table 3 compares Dice scores and Hausdorff distance between the various methods on the ISLES2018 dataset. The USSLNet is currently the state-of-the-art network for CT perfusion. Our method improves the mean Dice score performance over the state-of-the-art method, USSLNet, by 3.3%. It improves the HD95 score performance over USSLNet by 12.5%, as depicted in Table 3. nnUNet performs reasonably well, outperforms SPiN and is comparable to the performance of the USSLNet in terms of Dice score. However, it has the worst HD95 score compared to SPiN and USSLNet. HUT also surpasses the performance of the other methods used on CT Perfusion, such as the ERFNet and UShape network. It has a gain of 12.1% of dice score over both methods. The hybrid Transformer-based network TransBTS and UNETR are not working well on this dataset, mainly because the amount used to train the network is limited. Even though they are hybrid systems, the networks do not train as efficiently as their CNN counterparts. CLCI-Net and X-Net are not working well with the CT perfusion dataset, yielding only 0.310 and 0.336 dice scores, respectively. SPiN, on the contrary, performs quite well even when used in this dataset, scoring 0.561 for the dice score. HUT gains about 7.1% of dice score over the SPiN network.

**Table 3:** Comparison between mean and standard deviation (in parentheses) of dice score, HD95 score, IoU, precision, and recall of the ischemic stroke lesion segmentation with CT perfusion multimodal dataset against state-of-the-art methods.

**Methods**	**Dice**	**HD95**	**IOU**	**Precision**	**Recall**	**Memory**	**Infer**
		**(mm)**				**usage (Mb)**	**time (ms)**
UNet3D	0.451	23.102	0.334	0.676	0.375	3,178	627
(Ronneberger et al., 2015)	(0.206)	(10.705)	(0.173)	(0.294)	(0.173)		
ERFNet	0.537	16.180	0.415	0.776	0.470	1,442	1360
(Romera et al., 2017)	(0.225)	(6.604)	(0.205)	(0.242)	(0.218)		
UShape	0.476	22.066	0.366	0.574	0.459	3,712	685
(Clerigues et al., 2019)	(0.253)	(15.751)	(0.216)	(0.270)	(0.291)		
USSLNet	0.582	16.987	0.451	0.689	0.597	1,680	153
(Jiang and Chang, 2022)	(0.205)	(15.675)	(0.192)	(0.107)	(0.271)		
TransBTS	0.439	23.564	0.308	0.677	0.367	3,502	582
(Wang et al., 2021a)	(0.207)	(14.298)	(0.202)	(0.162)	(0.239)		
UNETR	0.469	20.648	0.337	0.503	0.496	7,054	635
(Hatamizadeh et al., 2022)	(0.229)	(13.146)	(0.208)	(0.258)	(0.252)		
CLCI-Net	0.310	24.127	0.221	0.546	0.253	–	353
(Yang et al., 2019)	(0.282)	(13.992)	(0.229)	(0.382)	(0.251)		
X-Net	0.336	21.246	0.239	0.506	0.286	–	317
(Qi et al., 2019)	(0.270)	(13.730)	(0.230)	(0.361)	(0.264)		
SPiN	0.561	19.119	0.423	0.563	**0.646**	646	377
(Wong et al., 2022)	(0.232)	(13.981)	(0.208)	(0.266)	(0.284)		
nnUnet	0.577	19.689	0.455	0.729	0.532	1470	141
(Isensee et al., 2021)	(0.222)	(13.439)	(0.210)	(0.232)	(0.237)		
HUT (ours)	**0.601**	**14.861**	**0.476**	**0.767**	0.551	1836	235
	(0.192)	(8.516)	(0.191)	(0.204)	(0.202)		

Inference memory usage and execution time per subject are included. Bold values highlight the best performing values.

We have also included the execution time per subject sample and memory usage during inference. nnUNet has the fastest execution time. SPiN has the lowest memory usage, but the execution time is slower than HUT. CLCI-Net and X-Net do not produce any numbers for memory usage due to the implementation in the older version of Tensorflow, which allocates full GPU memory during the inference.

**Figure 6:**
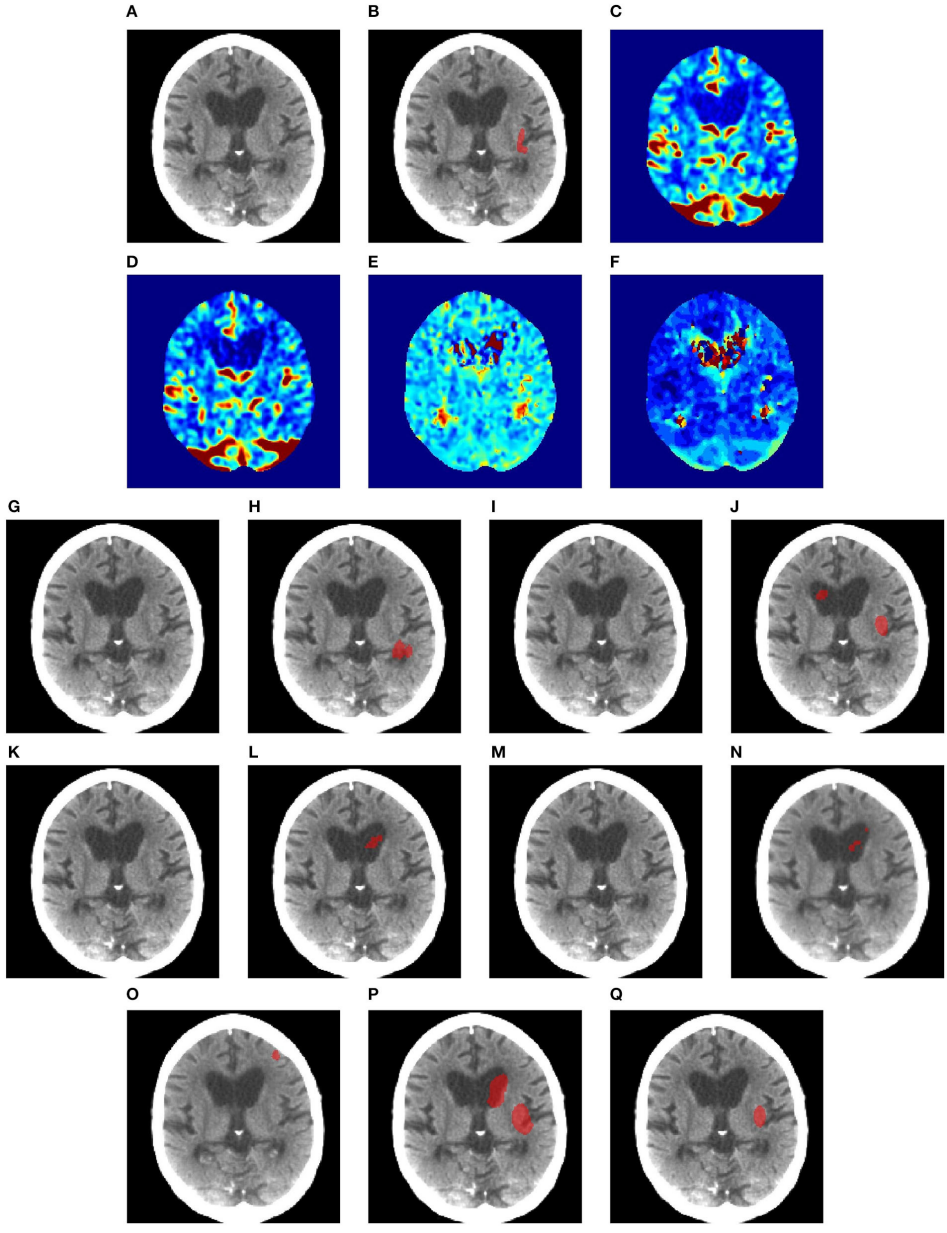
Illustration of lesion segmentation of representative subject 26 based on various methods. **(A)** CT scan, **(B)** ground truth, **(C)** CTP CBF, **(D)** CTP CBV, **(E)** CTP MTT, **(F)** CTP TMAX, **(G)** UNet3D, **(H)** ERFNet, **(I)** UShape, **(J)** USSLNet, **(K)** TransBTS, **(L)** UNETR, **(M)** CLCI-Net, **(N)** X-Net, **(O)** SPiN, **(P)** nnUnet, **(Q)** HUT.

**Figure 7:**
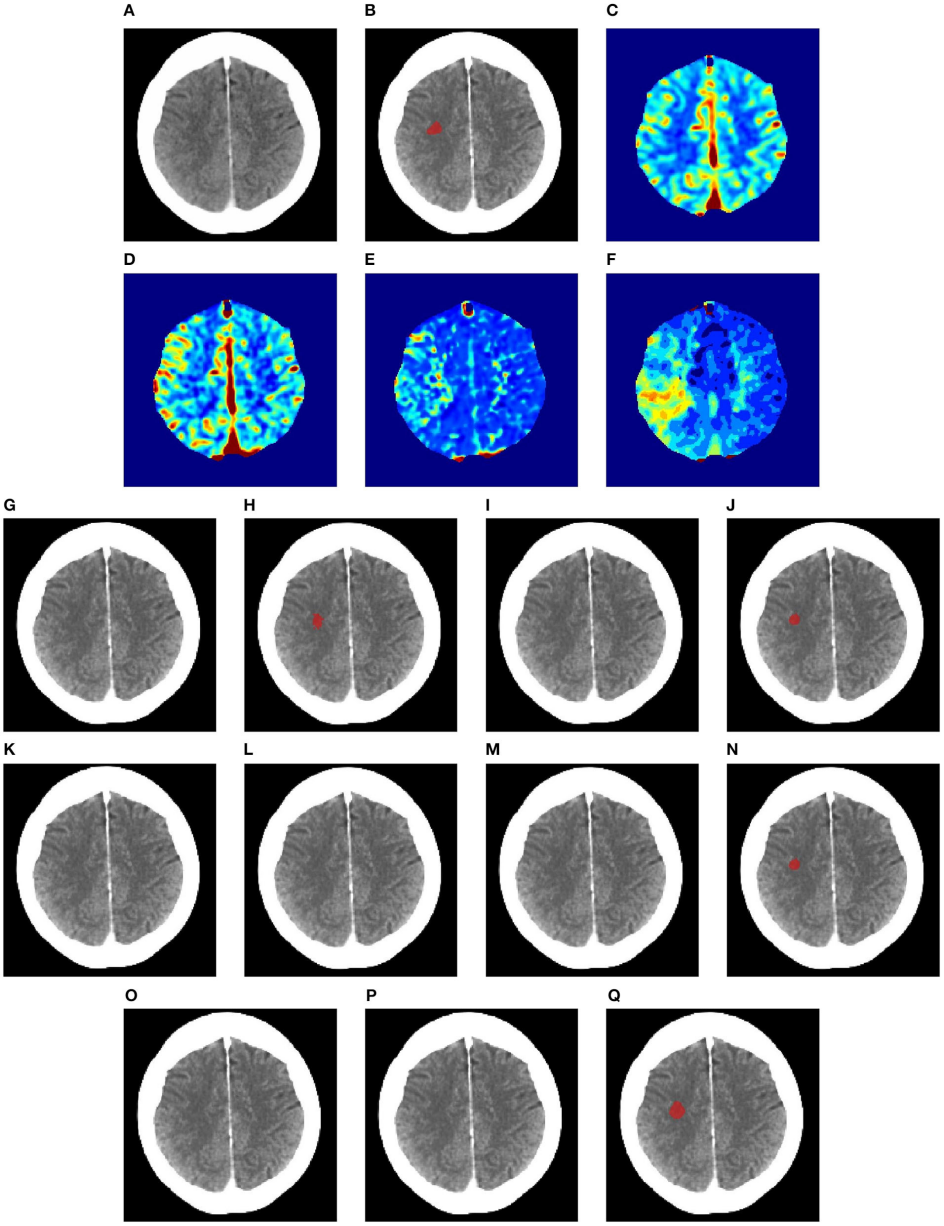
Illustration of lesion segmentation of representative subject 63 based on various methods. **(A)** CT scan, **(B)** ground truth, **(C)** CTP CBF, **(D)** CTP CBV, **(E)** CTP MTT, **(F)** CTP TMAX, **(G)** UNet3D, **(H)** ERFNet, **(I)** UShape, **(J)** USSLNet, **(K)** TransBTS, **(L)** UNETR, **(M)** CLCI-Net, **(N)** X-Net, **(O)** SPiN, **(P)** nnUnet, **(Q)** HUT.

**Figure 8:**
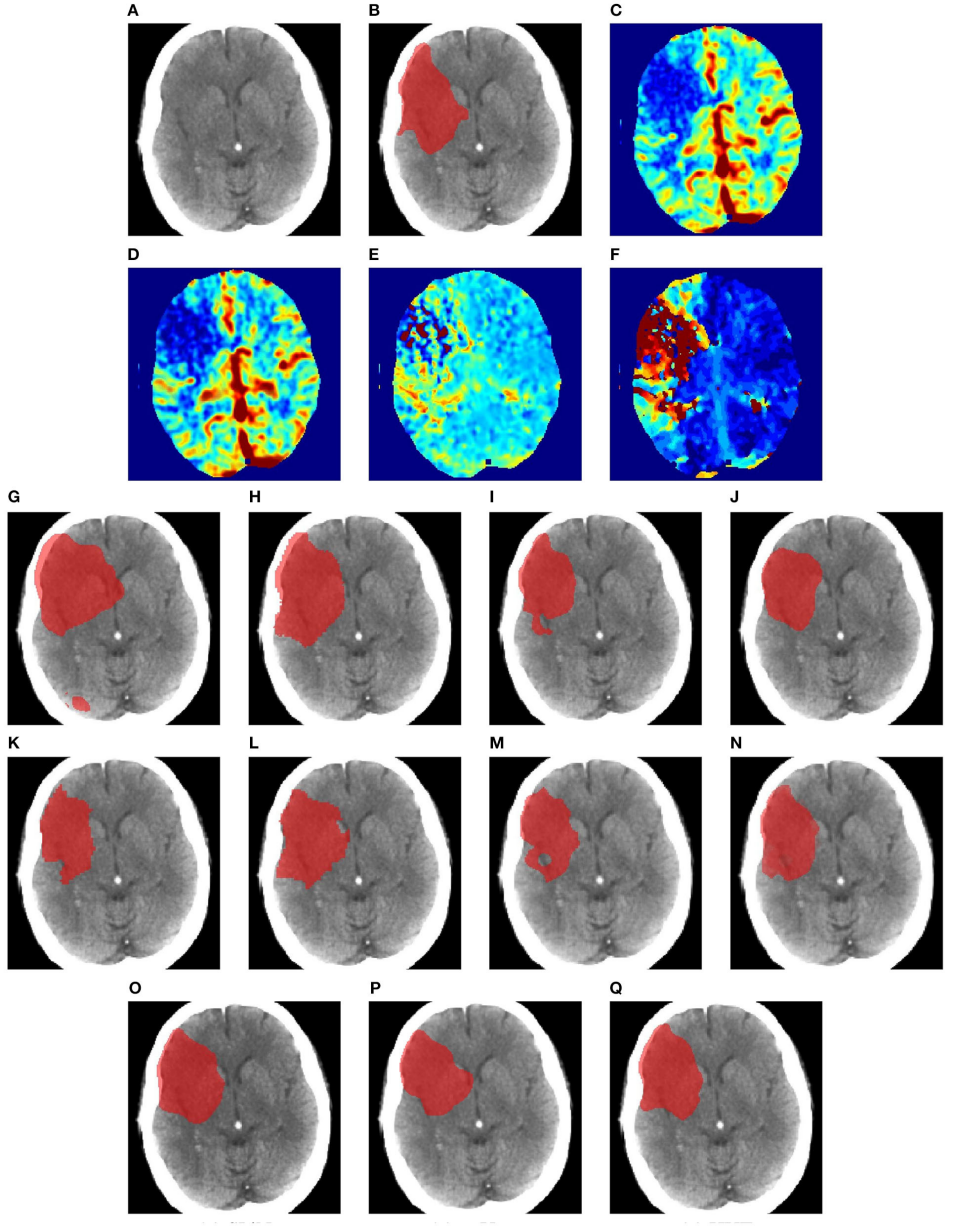
Illustration of lesion segmentation of representative subject 70 based on various methods. **(A)** CT scan, **(B)** ground truth, **(C)** CTP CBF, **(D)** CTP CBV, **(E)** CTP MTT, **(F)** CTP TMAX, **(G)** UNet3D, **(H)** ERFNet, **(I)** UShape, **(J)** USSLNet, **(K)** TransBTS, **(L)** UNETR, **(M)** CLCI-Net, **(N)** X-Net, **(O)** SPiN, **(P)** nnUnet, **(Q)** HUT.

Figures 6–8 illustrate the visual representations of the lesion segmentation using various methods. The first image is the measured CT scan. The second is the ground truth. Figures 6C–F, 7C–F, 8C–F are the CT perfusion images taken 8 h after the contrast agent is injected into the patient's bloodstream.

In Figure 6, we observe that the exact detection of the lesion is difficult, even through the perfusion map. The lesion is not visible on the CT scan. The perfusion maps of MTT and Tmax provide subtle information about the lesion. The methods, namely, UNet3D, Ushape, TransBTS, and CLCI-Net, cannot determine the lesion's location. UNETR and X-Net detect the same but the wrong location of the lesion. SPiN also highlights a false segmentation. ERFNet is close but not exact. USSLNet and nnUNet have one true positive and one false positive detection. It is unlikely that a lesion occurs in the ventricle. HUT is the only method which correctly detects the location of the lesion.

In Figure 7, the perfusion maps indicate that the lesion will likely appear on the left side. From the experiments, the methods that successfully detect the right location of the lesion are ERFNet, USSLNet, X-Net, and HUT. However, ERFNet produces fewer overlapping areas of segmentation with the ground truth. USSLNet and X-Net are smaller but accurate, whereas HUT covers a larger but accurate area.

In Figure 8, we illustrate the visual outlining produced by the methods when the lesion is large. The dice scores for most methods are high. However, some methods produce a better outline of the lesion than others. For instance, X-Net produces one of the better outlines, while UNet3D produces an over-enlarged area and a false positive at the bottom. nnUNet and USSLNet are more conservative in the detection of the lesion. HUT produces a most compelling outline closer to the ground truth.

### 3.3 Ablation study

#### 3.3.1 With MRI (T1-w) dataset

The results of the ablation study are shown in Table 4, in which we compare the performance of the baseline method by adding various components. The baseline method uses cross-entropy loss as the training objective. It excludes the self-supervised CLS training (*SS*) at the output of the cross-transformer's projection header of the CLS tokens by default. As for the ablation study on the ATLASR12 dataset, the proposed baseline model performs with a dice score of 0.720 and an HD95 of 13.64 mm, which still performs better than SPiN. The baseline model gains a dice score of 0.98% over the architecture without the VTS.

**Table 4:** Ablation study performance with mean dice score and HD95 score (in mm) of the ischemic stroke small lesion segmentation (AtlasR12) for HUT.

**Methods**	**Dice**	**HD95**	**IOU**	**Precision**	**Recall**
HUT (Baseline)	0.720	13.639	0.579	0.785	0.700
HUT without VTS	0.713	14.294	0.568	0.767	0.707
Baseline + *SS*	0.734	10.465	**0.601**	0.698	**0.801**
Baseline + *SS* + Focal loss	0.732	11.175	0.601	0.795	0.698
Baseline + *SS* + Dice loss	0.699	12.935	0.557	0.782	0.684
Baseline + *SS* + Balancing	**0.737**	**10.335**	0.598	**0.825**	0.706

Bold values highlight the best performing values.

Adding soft dice loss and self-supervised (*SS*) CLS training to the baseline causes a decline in performance. Soft dice loss (Milletari et al., 2016) is a loss function that alleviates the class imbalance issue by appropriately computing the difference between unity and the dice score. With focal loss and *SS*, the dice score improves to 0.732. A focal loss (Lin et al., 2017) is another loss function that tries to address the class imbalance in segmentation. The focal loss function down-weight the loss contributed by the easy examples by a modulating factor. Therefore, the loss for the harder examples will be relatively higher.

On the contrary, the model performs optimally using cross-entropy loss with a weighting of 0.15 for the background and 0.85 for the foreground. The model performs slightly worse than optimal without this weighting or balancing component. Weighting (Ronneberger et al., 2015) also mainly addresses the imbalance issue of the datasets as, in most cases, the portion of the background dominates the amount of the class label (lesion). It exerts more emphasis on the class label rather than the background. Therefore, the proposed architecture of HUT is best trained using the weighted cross-entropy loss function in all of these examples to address the class imbalance problem. We note that the class imbalance issue is closely related to the ability to detect a very small lesion in these ablation studies.

#### 3.3.2 With CT perfusion (CTP) dataset

For the CT perfusion (CTP) experiment, we adapted the ISLES2018 dataset, which contains only a few slices per subject. Therefore, we attempted to utilize the 2D slices or a chunk of scans for the training and testing instead. We compared the differences with and without the vision transformer, the advantage of self-supervision of the CLS token during the training and the differences between using slices and a chunk of slices for the training and inference.

As observed from Table 5, the baseline without the self-supervised training gains 4% of the dice score when the vision transformer is not incorporated into the system. The amount of self-supervised training also plays a part in the performance of the HUT system. The system's performance degrades when the λ_*SS*_ factor is set to 1. We observe that the training converges much faster if the value is higher at the expense of the peak performance. An empirical value of 1e-5 for λ_*SS*_ leads to the best performance.

**Table 5:** Ablation study performance with mean dice score and HD95 score (in mm) on the ISLES2018 dataset for HUT.

**Methods**	**Dice**	**HD95**	**IOU**	**Precision**	**Recall**
HUT (Baseline)	0.589	14.947	0.463	0.715	0.566
HUT without VTS	0.566	18.827	0.438	0.696	0.534
Baseline + *SS* with λ_*SS*_ = 1.0	0.545	19.734	0.421	0.760	0.460
Baseline + *SS* with λ_*SS*_ = 1*e*−5	**0.601**	**14.860**	**0.476**	**0.767**	**0.551**
Baseline + *SS* with λ_*SS*_ = 1*e*−5 and CHUNK of 3	0.584	19.495	0.469	0.701	0.549

Bold values highlight the best performing values.

We also compare the use of chunks of slices to train and predict. However, due to the nature of the dataset, the best performance is still obtained from the training using the slice-by-slice approach.

## 4 Discussion and conclusion

In segmenting ischemic strokes from T1-weighted MRI and CT perfusion scans, we used a hybrid U-Net and a cross-resolution transformer called the Hybrid UNet Transformer (HUT). The HUT network combines the UNet and the transformer to improve the task of the ischemic stroke segmentation from MRI and CT perfusion images. The network consists of parallel UNet and Transformer stages, leveraging the advantage of the inductive bias of image identification of the CNN layers and the pros of capturing global dependencies of image patches in the transformer. The cross-resolution transformer generates two different resolutions, which are then combined with U-Net's skip connections. We found that using two transformers, one for small patches and another for larger patches, followed by the cross transformer, helps improve performance with additional self-supervised learning. We employed CLS tokens for self-supervised learning and generated attention maps for the lower layers of the decoder.

There are several reasons that HUT has surpassed the performance of state-of-the-art methods in both MRI and CTP datasets. It is designed to address the local and long-range correlations between the patches, and it exceeds the capabilities of the current methods using transformers, UNet, and CNN for medical image segmentation by a considerable margin. The output of the VTS attends to information at various resolutions. The final output of the CLS tokens in VTS facilitates self-supervised learning with small and large patch transformers. It improves performance when datasets are small. The self-supervised training we incorporated does not require additional data from other datasets. The performance gained from the introduction of the transformer is helped by the self-supervised training of the CLS tokens with a faster rate of convergence. We have shown the advantages of the VTS with self-supervised training in the ablation studies. As a result, HUT gains a 4.84 and 41% improvement of dice score and HD95 score, respectively, over the SPiN in the single-modality MRI segmentation. It improves over USSLNet on multi-modality segmentation by 3.3% in the Dice score and 12.5% in the HD95 score.

However, despite a gain in overall performance, HUT has a higher precision but a lower recall in lesion segmentation. It shows an under-segmentation of the method on the ATLASR12 and ISLES2018 dataset, which has more small anomalies and subtle perfusion information to segment. Future work will investigate the possibility of gaining a higher recall while maintaining high precision by enhancing the networks and loss functions.

## Data availability statement

The datasets presented in this study can be found in online repositories. The names of the repository/repositories and accession number(s) can be found in the article/supplementary material.

## Author contributions

WS: Writing—original draft. JR: Writing—original draft.
